# Link-based influence maximization in networks of health promotion professionals

**DOI:** 10.1371/journal.pone.0256604

**Published:** 2021-08-25

**Authors:** Maurits H. W. Oostenbroek, Marco J. van der Leij, Quinten A. Meertens, Cees G. H. Diks, Heleen M. Wortelboer

**Affiliations:** 1 Center for Nonlinear Dynamics in Economics and Finance (CeNDEF), Amsterdam School of Economics, University of Amsterdam, Amsterdam, The Netherlands; 2 Tinbergen Institute, Amsterdam, The Netherlands; 3 The Leiden Institute of Advanced Computer Science (LIACS), Leiden University, Leiden, The Netherlands; 4 Netherlands Organisation for Applied Scientific Research (TNO), Zeist, The Netherlands; 5 Statistics Netherlands, The Hague, The Netherlands; 6 Congregation of the Blessed Sacrament, Bruxelles, Belgium; Unviersity of Burgundy, FRANCE

## Abstract

The influence maximization problem (IMP) as classically formulated is based on the strong assumption that “chosen” nodes always adopt the new product. In this paper we propose a new influence maximization problem, referred to as the “Link-based Influence Maximization Problem” (LIM), which differs from IMP in that the decision variable of the spreader has changed from choosing an optimal seed to selecting an optimal node to influence in order to maximize the spread. Based on our proof that LIM is NP-hard with a monotonic increasing and submodular target function, we propose a greedy algorithm, GLIM, for optimizing LIM and use numerical simulation to explore the performance in terms of spread and computation time in different network types. The results indicate that the performance of LIM varies across network types. We illustrate LIM by applying it in the context of a Dutch national health promotion program for prevention of youth obesity within a network of Dutch schools. GLIM is seen to outperform the other methods in all network types at the cost of a higher computation time. These results suggests that GLIM may be utilized to increase the effectiveness of health promotion programs.

## Introduction

The importance of (cost)effective community-based health promotion programs has become increasingly relevant as the world’s health care system and our society have become stressed by a significant increase in the global level of lifestyle related health problems. In the WHO European Region, noncommunicable diseases (NCDs), such as cancers, cardiovascular diseases, chronic obstructive pulmonary diseases and diabetes, are the leading cause of death, disease and disability [1]. One of the major risk factors for NCDs is overweight or obesity, an increasing proportion of children and adults are currently living with [2]. Because budgets in the public health domain are limited, the need for cost-effective health promotion programs is high and alternative approaches to improve the effectiveness of these programs are crucial. Computational methods developed in other fields, such as economics and sociology, can provide new approaches for systematic and in-depth understanding of alternative maximization of influence on incorporating a healthy lifestyle.

Community-based interventions are promising for health promotion and disease prevention but so far their potential is not fully realized [3]. It has been shown that health behavior can be influenced through social networks [4, 5]. Therefore, health promotion programs may be more effective if they are targeting the full network instead of only those observed. One could use the topological structure of the network to target the most effective interventions, as Christakis and Fowler [4] suggested. The main aspect of an intervention is the content of the intervention. However, also the amount of participation of the intervention is crucial, hopefully leading to more effective and lasting behavioral change.

The participation rate depends on the structure of the network in terms of clustering and the strength of ties [5, 6] and on where in the network the participation starts; Van der Leij [7] argues that policymakers could design social networks to influence the spread. Here we focus on how a network can be used to maximize the participation of either ‘individuals’, ‘institutions’ or ‘communities’ and the relationships between these participants in health promotion programs.

### Current literature

#### Social contagion and diffusion models

How behavior and information spreads through social contacts (social contagion) has been the focus of research for decades [6, 8–10]. Adoption of a product can be simulated using diffusion models resembling social contagion. In diffusion models it is assumed that the diffusion of the product starts at a certain node in the network, called the seed. In the independent cascade (IC) model, one assumes a probabilistic model in which a freshly activated node has a particular probability of activating any of its neighbours. This stochastic character handles the uncertainty regarding which people will decide to participate. In the threshold model, one assumes that every node has a certain influence on its neighbours. If a sufficient number of neighbours already participate, it will participate as well. Granovetter [11] introduced this model based on several examples, which all rely on the cooperative nature of people. It was found that people are more willing to participate if more of their neighbours already participate. This mechanism can be attributed to the product being either more effective, or more reliable or less risky. Multiple variations of these models have been proposed to capture different dynamics, such as the weighted cascade model, the general threshold model and the linear threshold model with colours [12].

#### The influence maximization problem

Domingos and Richardson [13] first addressed the problem of maximizing the diffusion of a product to as many people as possible as a fundamental algorithmic problem, called the influence maximization problem (IMP). In this problem it is assumed that a immutable social network exists, consisting of nodes and edges, while some external party wishes to market a product in this social network. The external party aims to sell its product to as many people as possible in the network, but they are restricted by a budget: it can introduce the product only to a limited number, *k*, of nodes, that will serve as the seeds of the diffusion process. The optimization problem in the IMP is then: to which *k* nodes should you introduce the product in order to maximize the spread of the product?

Kempe, Kleinberg and Tardos [14] further formalized the IMP and evaluated the properties of the spread as a function of the seeds, denoted by *σ*(⋅). They showed that the IMP is a nondeterministic polynomial time (NP)-hard problem. Since IMP is an NP-hard problem, there exists no algorithm that can find the optimal *k* nodes within polynomial time.

A commonly-used method to provide close-to-optimal solutions in NP-hard problems is the greedy algorithm. The greedy algorithm iteratively selects nodes until the budget of *k* nodes is satisfied. Within each iteration the greedy algorithm chooses the node that gives the best improvement of the target function. The greedy algorithm is known to perform well only under specific circumstances. Nemhauser, Wolsey and Fisher [15] showed that the solution provided by a greedy algorithm performs within (1 − 1/*e*) ≈ 0.63 of the optimal solution, if the target function is submodular. Submodularity of a set function refers to the property of diminishing returns, i.e. that the difference in the increase in the target function made by adding an element to the set decreases when the size of the input set increases.

Kempe, Kleinberg and Tardos [14] showed that the target function of IMP, i.e. the spread as a function of the chosen seeds, is both submodular and monotonically increasing under the independent cascade model. They stated that *σ*(⋅) is not necessarily submodular in the linear threshold model if the thresholds are assumed to be fixed [14]. They showed that the IMP target function of spread *σ*(⋅) is submodular and monotonically increasing in the linear threshold model under a uniform [0, 1] random threshold distribution. They then propose a greedy algorithm to optimize IMP in both the independent cascade model and the linear threshold model.

#### Influence estimation algorithms and optimization algorithms

Since the greedy algorithm as proposed by Kempe, Kleinberg and Tardos [14] uses the increase in influence *σ*(⋅) to choose nodes, the influence must be calculated for each potential node to use this algorithm. There are several methods of estimating the influence. Kempe, Kleinberg and Tardos [14] estimated the influence by using Monte Carlo (MC) simulations. Chen, Yuan and Zhang [16] showed that influence computation in threshold models is #P-hard in general graphs. Additionally, they derived a closed-form equation for calculating the estimated spread. However, since counting the number of simple paths is #P-hard, this calculation is computationally not scalable to larger networks, for which it becomes infeasible. They show that computing the influence in directed acyclic graphs (DAG) can be performed in linear time.

The recent literature focuses on finding efficient algorithms to estimate the spread and efficient optimization algorithms, and have led to, among other, the following results and adjusted optimization algorithms under the linear threshold model. It was observed that the influence of nodes quickly diminishes during diffusion in linear threshold models in many real-world networks [16, 17]. Lu et al. [18] devises the IMT algorithm that uses this characteristic to estimate the influence accurately and further provides an optimization algorithm. Goyal, Lu and Lakshmanan [17] developed the algorithm SIMPATH which efficiently estimates influence and optimizes the spread. Other efficient optimization algorithms are CELF++ [19] and LDAG [16]. Recent advances using the Reverse Influence Sampling (RIS) framework have led to nearly optimal optimization times in the IMP [20–22].

### Unsuitability IMP

Our main motivation lies in optimizing the spread of a health promotion program. The setting of a health promotion program may differ from that of social networks in which product adoption takes place. Health promotion programs are often implemented in institutions and require investment. We argue that the influence maximization problem as originally proposed does not align with the premise of optimizing the spread of a health promotion program for the following two reasons.

First, in the classical IMP one assumes that if a new seed is chosen, this seed will be activated with complete certainty. Participation in health promotion programs often involves time investment and active commitment, besides financial investment. Therefore, it is highly unlikely that every chosen node will participate in the setting of health promotion programs. We denote this shortcoming as the *persuasion problem*.

Second, the classical IMP optimizes the spread from a different perspective than the spreaders of health promotion programs. The classic IMP optimizes from the perspective of an external party: a company that is not part of the social network chooses starting points from which their product diffuses. In contrast, in health promotion networks the initiator of the program is often part of the network itself and tries to convince his peers to participate, labeled as the *perspective problem*. This discrepancy has two consequences. The first consequence is that since the initiator of the health promotion program is part of the network, there may already be some connections through which he or she influence peers, while the external party in the classic IMP does not have any influence before optimization. The second consequence is that in the classical IMP the external party has no influence on the structure of the network. On the other hand, the initiator of a health promotion program can influence the structure of the network by making new connections. We next argue how a link-based approach solves these two problems.

### Link-based influence maximization problem

We propose a new optimization problem for the maximization of spread of health promotion programs using an alternative target function that solves the persuasion and perspective problem as follows. The shortcomings of IMP are addressed by changing the decision variable of the optimization problem from choosing an optimal seed to selecting an optimal node to influence, which can be considered as creating a new link or connection to that particular node. Accordingly, we refer to this problem as the “Link-based Influence Maximization Problem” (LIM).

Creating new links to nodes solves the persuasion problem, because it creates paths to influence nodes, but does not assume certain participation of the nodes connected to. Furthermore, creating new links is a decision that can only be taken from the perspective of the health promotion program spreader, thus solving the perspective problem. Making new connections requires time investment, but time is limited. The restriction in time is analogous to a budget of *k* connections that can be made. In short, LIM centers around the question: to which *k* nodes does the spreader need to connect to maximize the spread?

The aim of this paper is to explore the characteristics of LIM. We limit ourselves to the framework of the *linear threshold* diffusion model for a number of reasons. First, health promotion programs typically require investment and require cooperation to be effective. Second, Centola [6] showed that people were more likely to adopt new behavior when they received social reinforcement. Third, the linear threshold model is based on endorsement through the network and is thus suitable for the premise of LIM. Future research is needed to explore the characteristics of LIM under different diffusion models.

Note that in practice creating links to new nodes, requires a personal effort and is mostly done within a comprehensible network, thus we assume that the networks in which LIM is applied are relatively small in contrast to the networks typically used in IMP and viral marketing, i.e. less than 500 nodes. Since we assume that LIM is applied in small networks, problems related to scalability and efficiency are less of an issue here. We therefore emphasize LIM as a conceptually new optimization problem, focusing on its characteristics and optimization performance here rather than on computing time For computing time considerations in the IMP context, see, e.g. [20–22]. We show that optimizing LIM could be utilized to increase the effectiveness of health promotion programs.

The remaining part of this paper is organized as follows. In Section “Link-based Influence Maximization” we focus on the characteristics of LIM in comparison to the classic IMP and show that LIM provides a different solution. We prove that the target function of LIM is submodular and monotonically increasing under the linear threshold model. Using these theoretical results, in Section “Performance of optimization algorithms applied to LIM” we propose a greedy algorithm based on influence estimation called GLIM, and compare the performance of GLIM to other optimization strategies using heuristics commonly used in social network analysis, and subsequently explore the performance of the greedy algorithm in different network types. In Section “Application: Dutch health promotion program” we apply the algorithm to a Dutch health promotion program in a network of public institutions of a single municipality in the Netherlands. Section “Discussion” discusses our results and concludes.

## Link-based influence maximization

The aim of influence maximization is to select nodes to influence, such that the spread of a product in a network of nodes is maximized given a cost constraint on the maximum number of nodes to select. The shortcoming of the classic IMP is the assumption that selected nodes will always adopt the offered product. We believe that a more realistic assumption is that selected nodes will only adopt the offered product with some node-specific probability. We assume that this probability depends on the relative influence of the spreader on the target node compared to the influence on the targeted node of other nodes in the network. We refer to the resulting optimization problem as LIM: the Link-based Influence Maximization Problem. The motivation for the name is that the action of the spreader trying to influence a node, can be viewed as creating a new (weighted) link between the two.

LIM deviates from the classical IMP on three points. First, in LIM, the decision variable is choosing the nodes, to which new links are made. Second, the spreader of the product is the seed of the diffusion, meaning that the seed of the diffusion process is fixed and the seed-node is the decision taker in optimizing the target function of total spread. Third, adding new edges to a target node affects the *relative* influence of other nodes to the target node.

In this chapter, we will formally introduce LIM and demonstrate by a simple example that it leads to a different optimal solution than the standard IMP. We point out that the optimization problem corresponding to LIM is NP-hard. However, we are able to prove that the set function corresponding to LIM is monotone and submodular and therefore, we may use a greedy algorithm to approximate the optimal solution.

### Formal definition of LIM

Consider a weighted directed graph *G* = (*V*, *E*, *w*) in which *V* is the set of nodes, *E* is the set of edges and *w* is a weight function. We allow for *G* to contain cycles. The weight function *w* assigns a positive weight *w*(*e*) ∈ (0, ∞) to each edge *e* ∈ *E*. The weight function models the influence that nodes have on each other. As we are interested in the *relative* (incoming) influence, we will normalize the incoming weights to sum up to 1. More precisely, we define for edge (*u*, *v*) from node *u* to node *v*:

$$\begin{array}{c} w_{n}\left(\left(u,v\right)\right)=\frac{w((u,v))}{\sum_{x\in N_{\text{in}}\left(v\right)}w\left(\left(x,v\right)\right)}, \end{array}$$ (1)

in which $N_{\text{in}}\left(v\right)$ denotes the in-neighborhood of *v*, $N_{\text{in}}\left(v\right)=\left\{x\in V:\left(x,v\right)\in E\right\}$. Note that the denominator in (1) is nonzero, because the weight *w*(*e*) is positive for all edges *e* ∈ *thesetofedgesE*. With slight abuse of notation, we will write *w*(*u*, *v*) instead of *w*((*u*, *v*)) from now on.

The framework of the linear threshold model is as follows. At the beginning of the diffusion process each node *v* chooses a threshold *θ*(*v*) from some continuous threshold distribution *μ*. The threshold distribution attains values between [0, 1] and a common choice is the uniform distribution on [0, 1]. The diffusion process can then be described recursively, as follows. Let *A*_*t*_ be the set of nodes that are activated at time *t* = 0, 1, 2, … as a result of the linear threshold process. In addition, define the boolean functions *z*_*t*_: *V* → {0, 1}, for *t* = 0, 1, 2, …, by setting *z*_*t*_(*v*) = 1 if node *v* has been activated at iteration *t* or before, and *z*_*t*_(*v*) = 0 if not. The relation between *A*_*t*_ and *z*_*t*_ is given by *A*_*t*_ = {*v*: *z*_*t*_(*v*) = 1}. At time *t* = 0 the initial seed (or *spreader*) *s* is activated, i.e., *A*_0_ = {*s*}. At each step *t* = 1, 2, 3, … new nodes can be activated. At every time step each node *v* evaluates if the *weighted* fraction of activated neighbours crosses the threshold. So *z*_*t*_(*v*) = 1 if the inequality

$$\begin{array}{c} \sum_{u\in N_{\text{in}}\left(v\right)}z_{t-1}\left(u\right)\cdot w_{n}\left(u,v\right)\geq \theta \left(v\right) \end{array}$$ (2)

is satisfied. The diffusion process continues until a time t* for which *z*_t*_(*v*) = *z*_t*−1_(*v*) for all *v* ∈ *V* or, equivalenty, *A*_t*_ = *A*_t*−1_. The total influence, or spread *σ*, is defined as the expected number of activated nodes at the end of the diffusion process, where the expectation is taken over the threshold distribution *μ*.

**Fig 1:**
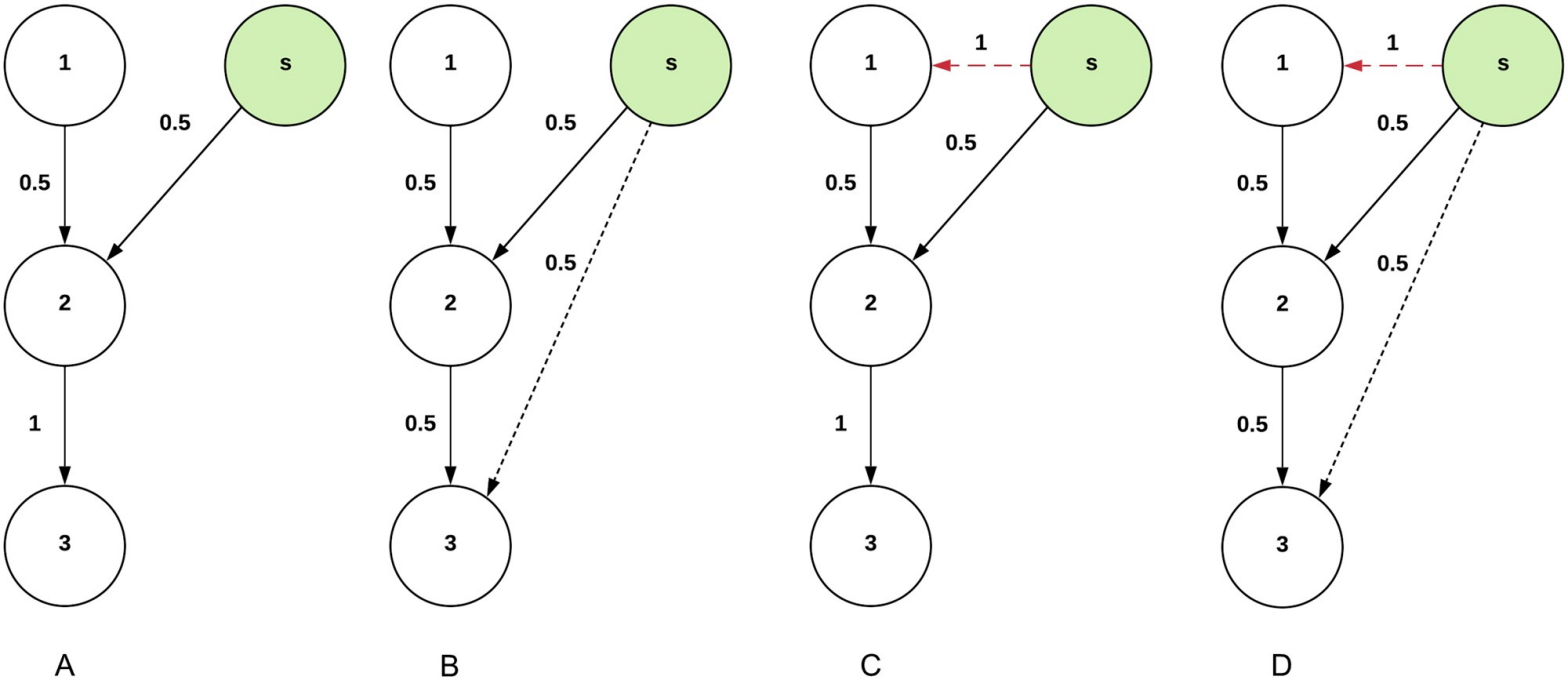
LIM: An example of how the addition of edges affects the network structure and weights of other edges. The circles represent nodes, in which the green circle is the spreader. The arrows represent edges and the numbers next to the arrows are the respective normalized weights. The striped and the red striped arrows depict new edges. A: Graph *G*. B: *G* + edge (*s*, 3). C: *G* + edge (*s*, 1). D: *G* + edges (*s*, 1), (*s*, 3).

In LIM, the spreader is assumed to be a single node *s* ∈ *V*. The initial action in the optimization process underlying the link-based influence maximization problem is to select a set *S* consisting of *k* nodes *v*_1_, …, *v*_*k*_,:(*s*, *v*_*i*_) ∉ *E* for which to add the edge (*s*, *v*_*i*_) to *E*. After selecting node *v*_*i*_ the weight function is updated such that *w*(*s*, *v*_*i*_) > 0, decreasing the *relative* influence of other nodes $x\in N_{\text{in}}\left(v_{i}\right)$ on *v*_*i*_ (an example of the addition of edges and its effect on the relative weight of other edges is shown in Fig 1).

The spread is a function of the chosen *k* nodes *v*_1_, …, *v*_*k*_ only. We denote the corresponding set function of spread by $\sigma :2^{V}\to N$. LIM can now be formulated as follows: given an integer *k*, which set of *k* nodes {*v*_1_, …, *v*_*k*_} maximize the spread *σ*({*v*_1_, …, *v*_*k*_})?

Finding the set *S* of cardinality *k* for which *σ*(*S*) is maximized is an NP-hard optimization problem, because it is a special case of the Hitting Set problem. Since LIM is an NP-hard problem, the optimal solution cannot be found within polynomial time. Nemhauser, Wolsey and Fisher [15] showed that greedy algorithms have an optimization guarantee in monotone submodular set functions, it provides a solution that gives a result at least (1 − 1/*e*) times the optimum in which *e* is the base of the natural logarithm. In subsection Monotonicity and Submodularity of the spread in LIM, we will prove that *σ* is a monotone submodular set function. Then, we may approximate the optimal solution to LIM by a greedy algorithm. In Section Performance of optimization algorithms applied to LIM, we propose a greedy algorithm similar to the algorithm proposed by Kempe, Kleinberg and Tardos [14]. We will now show that optimizing LIM provides different solutions than the classic IMP.

**Fig 2:**
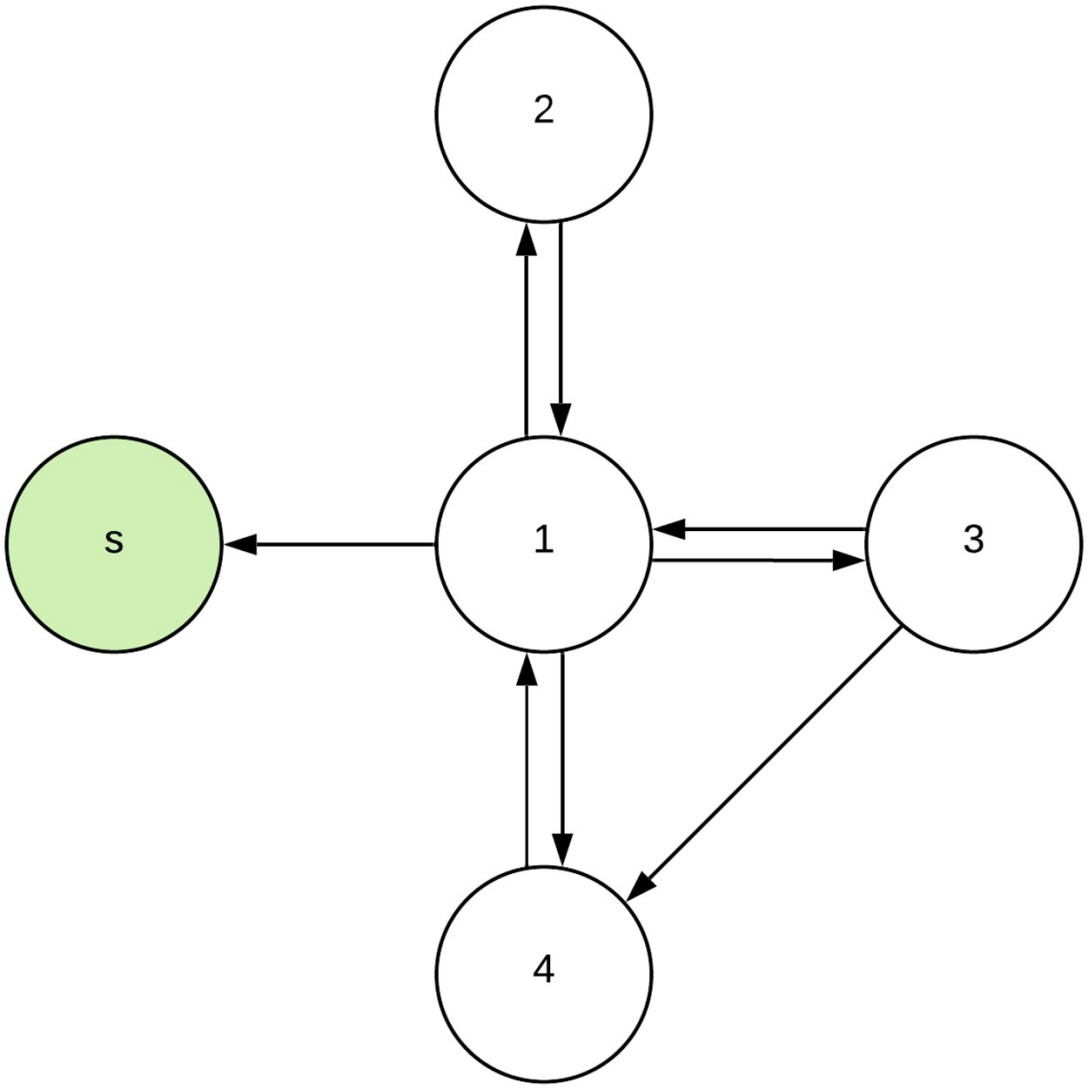
Example network. The circles represent nodes, in which the green circle is the spreader. The arrows represent edges.

To show that LIM and IMP result in different solutions, we include a simple example. Consider the graph of 5 nodes, {*s*, 1, 2, 3, 4} as depicted in Fig 2. The weights of all edges are identical, say equal to 1, so that the normalised weights are the reciprocal of the target node’s in degree, $w_{n}\left(e_{v}\right)=1/d_{v}^{in}$. Now, if we set the budget constraint to 1 node, IMP will select node 1 to target, while LIM will select node 3 to target, see Appendix S1 Appendix for the computations. IMP selects node 1 because node 1 has the largest influence on the entire network and because IMP assumes that the target node is always convinced (the persuasion problem). However, node 1 is also influenced by many other nodes, so in reality it might be more difficult to convince node 1 to join the program in the first place. On the other hand, node 3 also has a relatively large influence on the network, whilst the influence of other nodes on node 3 is limited. Therefore, we believe that node 3 might be more easily convinced to join the program. Hence, under LIM targeting node 3 would contribute more to the adaption of the program by the network than targeting node 1. Furthermore, the calculation shows that the expected increase in spread after targeting nodes under LIM is lower than under the classic IMP, for all nodes.

### Monotonicity and submodularity of the spread in LIM

In this subsection, we will prove that the set function of spread *σ* is a monotone submodular set function. It amounts to proving two properties, (A) monotonicity and (B) submodularity:

A. For any *S* ⊂ *V* and any *x* ∈ *V*, (*s*, *x*) ∉ *E*: *σ*(*S* ∪ {*x*}) ≥ *σ*(*S*).
B. For any *S* ⊂ *V* and any two *x*, *y* ∈ *V*, (*s*, *x*), (*s*, *y*) ∉ *E*:
  *σ*(*S* ∪ {*x*}) − *σ*(*S*) ≥ *σ*(*S* ∪ {*x*, *y*}) − *σ*(*S* ∪ {*y*}).

Before we present the proof of the two properties, we introduce some additional notation which is required to keep track of the changes made to *E* and to *w*_*n*_ when edges of the form (*s*, *v*) (where *s* ∈ *V* is the spreader and seed, and *v* is another node in *V*) are added. From now on, *G*, *E* and *w*_*n*_ will only be used to denote the graph with the initial set of edges (i.e., before links are added) and the corresponding normalized weight function, respectively. Then, for a selected subset *S* ⊂ *V*, we define

$$\begin{array}{c} E^{S}≔E\cup \underset{v\in S}{⋃}\left\{\left(s,v\right)\right\}. \end{array}$$

The updated weight function *w*^*S*^ is the extension of *w* from *E* to *E*^*S*^, assigning positive value to new edges, i.e. *w*^*S*^(*e*) = *w*(*e*) for *e* ∈ *E* and *w*^*S*^(*s*, *v*) > 0 for *v* ∈ *S*. Finally, we define $w_{n}^{S}\left(u,v\right)$ for (*u*, *v*) ∈ *E*^*S*^ by

$$\begin{array}{c} w_{n}^{S}\left(u,v\right)=\frac{w^{S}\left(u,v\right)}{\sum_{x\in N_{\text{in}}^{S}}w^{S}\left(x,v\right)}, \end{array}$$

in which $N_{\text{in}}^{S}\left(v\right)=\left\{x\in V:\left(x,v\right)\in E^{S}\right\}$ is the in-neighborhood of a node *v* considering the extended set of edges *E*^*S*^. The resulting weighted directed graph is denoted by *G*^*S*^ = (*V*, *E*^*S*^, *w*^*S*^).

The spread, resulting from adding edges, is the expected number of activated nodes at the end of the diffusion process in the new graph, where the expectation is taken over the threshold distribution *μ*, as a function of the chosen *k* nodes *v*_1_, …, *v*_*k*_ only:

$$\begin{array}{c} \sigma \left(S\right)=E[|A_{t^{*}}||G^{S},s,\mu ]. \end{array}$$

We are now ready to prove the monotonicity of the set function *σ*.

**Theorem 1**. *The spread σ is a monotone set function under the assumption that the threshold distribution is the uniform distribution on* [0, 1].

*Proof.* Consider a positively weighted directed graph *G* = (*V*, *E*, *w*) with a spreader node *s* ∈ *V*. Let *S* ⊂ *V* be a subset of *V* and let *x* ∈ *V*. We will show that *σ*(*S* ∪ {*x*}) − *σ*(*S*) ≥ 0.

Chen, Yuan and Zhang [16] proved the following identity under the assumption that the threshold distribution is the uniform distribution on [0, 1]:

$$\begin{array}{c} \sigma \left(S\right)=E[|S_{t^{*}}\left||G,s,\mu \right]=\sum_{\pi \in P_{s}^{S}}\;\prod_{e\in \pi }w_{n}^{S}\left(e\right). \end{array}$$ (3)

In this equation, $P_{s}^{S}$ is the set of all simple paths in *G*^*S*^ = (*V*, *E*^*S*^, *w*^*S*^) that start in node *s*. When node *x* ∈ *V* is added to the set *S*, two things change in the computation of *σ*. First, the set $P_{s}^{S\cup \{x\}}$ contains new paths that are not in $P_{s}^{S}$ (i.e., starting with edge (*s*, *x*)), so we sum over a larger set. Second, for all paths $\pi \in P_{s}^{S}$ that already hit *x* at some point, say, *e* = (*u*, *x*) ∈ *π* for some *u* ∈ *V*, the relative influence of that node *u* on *x* decreases as a result of the assigned weight to edge *w*(*s*, *x*) > 0. The normalized weight changes as follows:

$$\begin{array}{cc} w_{n}^{S\cup \{x\}}\left(u,x\right) & =\frac{w(u,x)}{\sum_{v\in N_{\text{in}}^{S\cup \{x\}}\left(x\right)}w\left(v,x\right)}=\frac{w(u,x)}{w\left(s,x\right)+\sum_{v\in N_{\text{in}}^{S}\left(x\right)}w\left(v,x\right)}\\ & =\frac{\sum_{v\in N_{\text{in}}^{S}}\left(x\right)w\left(v,x\right)}{w\left(s,x\right)+\sum_{v\in N_{\text{in}}^{S}\left(x\right)}w\left(v,x\right)}\cdot w_{n}^{S}\left(u,x\right). \end{array}$$ (4)

We note that the contribution to *σ* of paths in $P_{s}^{S}$ that do not hit *x* will not change after adding edge (*s*, *x*) to *E*^*S*^. Hence, it follows that the difference *σ*(*S* ∪ {*x*}) − *σ*(*S*) equals

$$\begin{array}{c} \sigma \left(S\cup \left\{x\right\}\right)-\sigma \left(S\right)=\sum_{\pi \in P_{s,x}^{S\cup \{x\}}}\;\prod_{e\in \pi }w_{n}^{S\cup \{x\}}\left(e\right)-\sum_{\pi \in P_{s,x}^{S}}\;\prod_{e\in \pi }w_{n}^{S}\left(e\right), \end{array}$$ (5)

in which $P_{s,x}^{S}\subset P_{s}^{S}$ is the set of paths in *G*^*S*^ starting at *s* and hitting *x* at some point. In general, we will write $P_{a,b}^{S}$ for the set of paths in *G*^*S*^ starting at node *a* and hitting node *b* at some point. Moreover, we will write ${\bar{P}}_{a,b}^{S}$ for the set of paths in *G*^*S*^ starting at *a* and ending at *b*. Now, to evaluate the right-hand side of Eq (5), the idea is to split the contribution to *σ* of a path in $P_{s,x}^{S}$ into the part before hitting *x* and the part starting at *x*. We then aggregate the contributions to *σ* of the paths before hitting *x* that can be concatenated with the path starting at *x*. When aggregating, we should be careful, as *G*^*S*^ may contain cycles. We then show that the contributions of the paths hitting *x* increase when (*s*, *x*) is added to *E*^*S*^.

We formalize the idea as follows. Consider the set $P_{x}^{S}$ of paths in *G*^*S*^ that start at node *x*. We will assume, without loss of generality, that $N_{\text{in}}^{S}\left(s\right)=\emptyset$. That assumption guarantees that paths in $P_{x}^{S}$ do not hit spreader node *s*. For any path $\rho \in P_{x}^{S}$, define the *output* of *ρ* as follows:

$$\begin{array}{c} U^{S}≔\left(\rho \right)\prod_{e\in \rho }w_{n}^{S}\left(e\right). \end{array}$$ (6)

We will explicitly include the empty path ∅ in $P_{x}^{S}$ and use the convention that an empty product equals 1, i.e., *U*^*S*^(∅) = 1. Next, define the set of all paths preceding $\rho \in P_{x}^{S}$ in $P_{s}^{S}$ as

$$\begin{array}{c} A_{x}^{S}\left(\rho \right)≔\left\{\phi \in {\bar{P}}_{s,x}^{S}:\phi \rho \in P_{s,x}^{S}\right\}, \end{array}$$ (7)

in which *ϕρ* denotes the concatenation of paths *ϕ* and *ρ*. Then, for $\rho \in P_{x}^{S}$, define the *input* of *ρ* as

$$\begin{array}{c} I^{S}\left(\rho \right)≔\sum_{\phi \in A_{x}^{S}\left(\rho \right)}\prod_{e\in \phi }w_{n}^{S}\left(e\right). \end{array}$$ (8)

Note that for some $\rho \in P_{x}^{S}$, the set $A_{x}^{S}\left(\rho \right)$ might be empty. In that case, we use the convention that an empty sum equals 0, i.e., *I*^*S*^(*ρ*) = 0 in that case (observe that it does not necessarily happen for the empty path ∅, as $A_{x}^{S}\left(\emptyset \right)={\bar{P}}_{s,x}^{S}$). In addition, note that an upper bound on *I*^*S*^(*ρ*) is the probability that *x* is activated under the assumed linear threshold model in graph *G*^*S*^ after activating node *s*, which is at most 1. It follows from the fact that this probability equals $\sum_{\phi \in {\bar{P}}_{s,x}^{S}}\prod_{e\in \phi }w_{n}^{S}\left(e\right)$ [14], and the fact that $A_{x}^{S}\left(\rho \right)\subset {\bar{P}}_{s,x}^{S}$. In particular, it implies that *I*^*S*^(*ρ*) ≤ 1 for any $\rho \in P_{x}^{S}$.

With the above notation introduced, it directly follows that

$$\begin{array}{c} \sum_{\pi \in P_{s,x}^{S}}\prod_{e\in \pi }w_{n}^{S}\left(e\right)=\prod_{\rho \in P_{x}^{S}}\left(\sum_{\phi \in A_{x}^{S}}\prod_{e\in \phi \rho }w_{n}^{S}\left(e\right)\right)=\prod_{\rho \in P_{x}^{S}}I^{S}\left(\rho \right)U^{S}\left(\rho \right). \end{array}$$ (9)

A similar expression holds for the first summation on the right-hand side of Eq (5). To compare the resulting expressions, we first note that $P_{x}^{S\cup \{x\}}=P_{x}^{S}$. Moreover, for each $\rho \in P_{x}^{S}$, it holds that *U*^*S*∪{*x*}^(*ρ*) = *U*^*S*^(*ρ*). Furthermore, for any path $\rho \in P_{x}^{S}$, the set *A*^*S*∪{*x*}^(*ρ*) is equal to the set *A*^*S*^(*ρ*) ∪ {〈*s*, *x*〉}. Using Eq (4), it follows that

$$\begin{array}{cc} I^{S\cup \{x\}}\left(\rho \right)-I^{S}\left(\rho \right) & =w_{n}^{S\cup \{x\}}\left(s,x\right)+\sum_{\phi \in A^{S}\left(\rho \right)}\prod_{e\in \phi }w_{n}^{S\cup \{x\}}\left(e\right)-I^{S}\left(\rho \right)\\ & =\frac{w(s,x)}{w\left(s,x\right)+\sum_{v\in N_{\text{in}}^{S}\left(x\right)}w\left(v,x\right)}+\\ & \;\frac{\sum_{v\in N_{\text{in}}^{S}\left(x\right)}w\left(v,x\right)}{w\left(s,x\right)+\sum_{v\in N_{\text{in}}^{S}\left(x\right)}w\left(v,x\right)}\cdot I^{S}\left(\rho \right)-I^{S}\left(\rho \right)\\ & =w_{n}^{S\cup \{x\}}\left(s,x\right)(1-I^{S}\left(\rho \right)). \end{array}$$ (10)

The conclusion is that

$$\begin{array}{c} \sigma (S\cup \{x\})-\sigma (S)=w_{n}^{S\cup \{x\}}\left(s,x\right)\cdot \sum_{\rho \in P_{x}^{S}}\left(1-I^{S}\left(\rho \right)\right)\cdot U^{S}\left(\rho \right), \end{array}$$ (11)

which is nonnegative because *I*^*S*^(*ρ*) ≤ 1. This concludes the proof.

We will now prove that the set function *σ* is submodular as well.

**Theorem 2**. *The spread σ is a submodular set function under the assumption that the threshold distribution is the uniform distribution on* [0, 1].

*Proof.* Consider a positively weighted directed graph *G* = (*V*, *E*, *w*) with a spreader node *s* ∈ *V*. Let *S* ⊂ *V* be a subset of *V* and let *x*, *y* ∈ *V*. We will show that

$$\begin{array}{c} \sigma (S\cup \{x\})-\sigma (S)\geq \sigma (S\cup \{x,y\})-\sigma (S\cup \{y\}). \end{array}$$

Assume, without loss of generality, that $N_{\text{in}}^{S}\left(s\right)=\emptyset$. Then, $P_{x}^{S\cup \{y\}}=P_{x}^{S}$ and $P_{x}^{S\cup \{x,y\}}=P_{x}^{S}$. To compute *σ*(*S* ∪ {*x*, *y*}) − *σ*(*S* ∪ {*y*}), we use Eq (11), the fact that $P_{x}^{S\cup \{y\}}=P_{x}^{S}$ and the fact that $w_{n}^{S\cup \{x,y\}}\left(s,x\right)=w_{n}^{S\cup \{x\}}\left(s,x\right)$:

$$\begin{array}{c} \sigma \left(S\cup \left\{x,y\right\}\right)-\sigma \left(S\cup \left\{y\right\}\right)=w_{n}^{S\cup \{x\}}\left(s,x\right)\cdot \sum_{\rho \in P_{x}^{S}}\left(1-I^{S\cup \{y\}}\left(\rho \right)\right)\cdot U^{S\cup \{y\}}\left(\rho \right). \end{array}$$ (12)

We compare *σ*(*S* ∪ {*x*}) − *σ*(*S*) with *σ*(*S* ∪ {*x*, *y*}) − *σ*(*S* ∪ {*y*}) by comparing the expressions on the right-hand sides of Eqs (11) and (12) term by term. To that end, we let $\rho \in P_{x}^{S}$ and we distinguish three cases.

In the first case, we assume that *y* ∈ *ρ*. Then, there exists a unique *u* ∈ *V*, *u* ≠ *s*, such that (*u*, *y*) ∈ *ρ*. From Eq (4) it then follows that $w_{n}^{S\cup \{y\}}\left(u,y\right)<w_{n}^{S}\left(u,y\right)$, which implies *U*^*S*∪{*y*}^(*ρ*) < *U*^*S*^(*ρ*). Moreover, *y* ∈ *ρ* implies that *y* ∉ *ϕ* for all *ϕ* ∈ *A*^*S*∪{*y*}^(*ρ*), and hence *I*^*S*∪{*y*}^(*ρ*) = *I*^*S*^(*ρ*). Thus, we obtain the inequality (1 − *I*^*S*∪{*y*}^(*ρ*)) ⋅ *U*^*S*∪{*y*}^(*ρ*) < (1 − *I*^*S*^(*ρ*))⋅*U*^*S*^(*ρ*).

In the second case, we assume that *y* ∉ *ρ* and *y* ∉ *ϕ* for all *ϕ* ∈ *A*^*S*∪{*y*}^(*ρ*). The first assumption implies that *U*^*S*∪{*y*}^(*ρ*) = *U*^*S*^(*ρ*) and the second assumption implies that *I*^*S*∪{*y*}^(*ρ*) = *I*^*S*^(*ρ*). Hence, we obtain (1 − *I*^*S*∪{*y*}^(*ρ*)) ⋅ *U*^*S*∪{*y*}^(*ρ*) = (1 − *I*^*S*^(*ρ*)) ⋅ *U*^*S*^(*ρ*).

In the third case, we assume that *y* ∉ *ρ*, but that there exists at least one path *ϕ* ∈ *A*^*S*∪{*y*}^(*ρ*) that contains *y*. The assumption *y* ∉ *ρ* implies that *U*^*S*∪{*y*}^(*ρ*) = *U*^*S*^(*ρ*). To compare *I*^*S*∪{*y*}^(*ρ*) with *I*^*S*^(*ρ*), we observe that we only need to consider the paths in *A*^*S*∪{*y*}^(*ρ*) that contain *y*. For those paths, we will split the corresponding terms in *I*^*S*∪{*y*}^(*ρ*) and *I*^*S*^(*ρ*) into the part before *y* and the part between *y* and *x*. To that end, recall that ${\bar{P}}_{a,b}^{S}$ is the set of paths in *G*^*S*^ from node *a* to node *b*. For any two paths $\xi \in {\bar{P}}_{y,x}^{S}$ and $\rho \in P_{x}^{S}$ we define the set

$$\begin{array}{c} B_{y,x}^{S}\left(\xi ,\rho \right)\left\{\zeta \in {\bar{P}}_{s,y}^{S}:\zeta \xi \in A_{x}^{S}\left(\rho \right)\right\}. \end{array}$$ (13)

If *ξρ* is not a simple path, we obtain $B_{y,x}^{S}\left(\xi ,\rho \right)=\emptyset$. It follows that

$$\begin{array}{cc} I^{S\cup \{y\}}\left(\rho \right)-I^{S}\left(\rho \right) & =\sum_{\phi \in A_{x}^{S\cup \{y\}}\left(\rho \right)}\prod_{e\in \phi }w_{n}^{S\cup \{y\}}\left(e\right)-\sum_{\phi \in A_{x}^{S}\left(\rho \right)}\prod_{e\in \phi }w_{n}^{S}\left(e\right)\\ & =\sum_{\xi \in {\bar{P}}_{y,x}^{S\cup \{y\}}}\sum_{\zeta \in B_{y,x}^{S\cup \{y\}}\left(\xi ,\rho \right)}\prod_{e\in \zeta \xi }w_{n}^{S\cup \{y\}}\left(e\right)-\sum_{\xi \in {\bar{P}}_{y,x}^{S}}\sum_{\zeta \in B_{y,x}^{S}\left(\xi ,\rho \right)}\prod_{e\in \zeta \xi }w_{n}^{S}\left(e\right). \end{array}$$ (14)

The set ${\bar{P}}_{y,x}^{S\cup \{y\}}$ equals ${\bar{P}}_{y,x}^{S}$ as noted before. The set $B_{y,x}^{S\cup \{y\}}\left(\xi ,\rho \right)$ equals $B_{y,x}^{S}\left(\xi ,\rho \right)\cup \left\{\langle s,y\rangle \right\}$. Then, similar to Eq (10), it holds for any $\xi \in {\bar{P}}_{y,x}^{S}$ that

$$\begin{array}{c} \sum_{\zeta \in B_{y,x}^{S\cup \{y\}}\left(\xi ,\rho \right)}\prod_{e\in \zeta \xi }w_{n}^{S\cup \{y\}}\left(e\right)-\sum_{\zeta \in B_{y,x}^{S}\left(\xi ,\rho \right)}\prod_{e\in \zeta \xi }w_{n}^{S}\left(e\right)=w_{n}^{S\cup \{y\}}\left(s,y\right)\cdot \left(1-J^{S}\left(\xi ,\rho \right)\right)\cdot U^{S}\left(\xi \right), \end{array}$$ (15)

in which $J^{S}\left(\xi ,\rho \right)=\sum_{\zeta \in B_{y,x}^{S}\left(\xi ,\rho \right)}\prod_{e\in \zeta }w_{n}^{S}\left(e\right)$, where the product only runs over edges in *ζ* but not over those in *ξ*. It is clear that *J*^*S*^(*ξ*, *ρ*) ≤ *I*^*S*^(*ξ*), which in turn is bounded from above by 1, as we noted in the proof of Theorem 1. The implication is that *I*^*S*∪{*y*}^(*ρ*) − *I*^*S*^(*ρ*) ≥ 0 and thus 1 − *I*^*S*∪{*y*}^(*ρ*) ≤ 1 − *I*^*S*^(*ρ*). We conclude that (1 − *I*^*S*∪{*y*}^(*ρ*)) ⋅ *U*^*S*∪{*y*}^(*ρ*) ≤ (1 − *I*^*S*^(*ρ*)) ⋅ *U*^*S*^(*ρ*).

We have shown that each term in the summation in Eq (12) is bounded from above by the corresponding term in the summation in Eq (11). This concludes the proof.

We have now proved that the target function of LIM is both monotonic increasing and submodular. Therefore as noted before, a greedy algorithm attains the optimization guarantee. In the next section we propose a greedy algorithm that optimizes LIM based on the estimated influence and compare it to other optimization algorithms.

## Performance of optimization algorithms applied to LIM

In Section “Link-based Influence Maximization”, we noted that LIM is a NP-hard problem and has a monotonically increasing submodular target function. Therefore, we propose a greedy algorithm to provide *k* links to maximize the total spread, denoted as GLIM. First, we will elaborate on GLIM. In the next subsection, we explore the performance of GLIM in comparison to alternative optimization strategies for LIM using basic network measures. The performance is measured in the resulting improvement in spread and in computation time. The performance of all optimization strategies will be explored in different network types, to identify in which network types using GLIM is most advantageous. Note that we will not compare optimizing LIM to IMP, as they are conceptually different optimization problems leading to distinct diffusion mechanics.

The algorithm we propose, GLIM, is similar to the algorithm proposed by Kempe, Kleinberg and Tardos [14]. The goal of the algorithm is to select a set of nodes *S* of size *k* to which seed *s* can connect to, to optimize the total spread. Nodes are added to set *S* iteratively by selecting the node that gives the highest estimated spread when connected to. The algorithm is shown in Table 1.

**Table 1:** GLIM: Greedy algorithm for edge selection in LIM.

0:	input: weighted directed graph *G* = (*V*, *E*, *w*), seed *s* and budget *k*
1:	Let S=⌀ is the set of new nodes to connect to and IL =⌀ the list of total influences of seed *s* when connected to node *v*
2:	Let *V*^*s*^ be the list of nodes *v* for which edges (*s*, *v*) not in *E*
3:	**while** |*S*| < *k* or $V^{s}=⌀$ **do:**
4:	**for** each node *v* in *V*^*s*^:
5:	use MC to estimate *σ*(*S* ∪ *v*)
6:	**end for**
7:	construct IL = {*σ*(*S* ∪ *v*_1_), …, *σ*(*S* ∪*v*_*n*_)}
8:	select v* = argmax (IL)
9:	*S* = *S* ∪ v*, *E*^*S*^ = *E*^*S*^ ∪ (*s*, v*), *V*^*s*^ = *V*^*s*^∖v*
10:	update *w*^*S*^, with $w\left(u,v^{*}\right)=1/d_{v^{*}}^{in}$ $\forall u\in N_{v^{*}}$
11:	**end while**
12:	**return** *S*

We estimate the potential increase in spread by including the edge in the network and subsequently estimating the spread with Monte Carlo (MC) simulation. Estimation spread using MC simulation works as follows: For a number of simulations, every simulation run thresholds *θ* are drawn from a random uniform [0, 1] distribution and then diffusion is simulated following the linear threshold model, as described in section Formal definition of LIM, until no further nodes can be activated. The spread is then the fraction of nodes that are activated at the end of the simulation run. The total spread is estimated by the average of spread over all simulations. For each node 1000 simulations are performed. A greedy algorithm using MC simulation has been known to be inefficient and is not scalable to larger networks. However, since we assume that LIM is applied in small networks, scalability poses no problem and GLIM can be used.

### Comparison of GLIM to other optimization algorithms

In sociology, degree and centrality-based measures are commonly used to measure the importance of nodes in networks [23]. Thus using these measures in optimization strategies is an obvious choice. Furthermore, the advantage of network measures is that the computation time is considerably lower than estimating the influence using with MC simulation in every iteration of the algorithm. In the particular case of LIM, we want to maximize spread, so we are interested in the influence that a node has on other nodes. Therefore, we compare GLIM, which is based on the influence estimation, to optimization algorithms based on the measures outdegree and right eigenvector centrality.

#### Alternative optimization algorithms

The optimization algorithms based on the network measures are also greedy algorithms. Instead of choosing the edge with the highest resulting estimated spread, we choose the edge connecting to the node with respectively the highest outdegree or right eigenvector centrality. The outdegree of a node is not affected by adding the edge connecting to it, so no recalculation is necessary during the algorithm. The eigenvector centrality does change after including edges, therefore, the eigenvector centrality will be updated after each iteration. The process of adding edges is repeated until the budget is satisfied or until there are no more edges available. Finally, all methods are compared to a baseline obtained by random edge selection.

#### Network types

The structure of a network has a direct influence on the diffusion process. The quality of solutions given by GLIM may differ for various network structures. Targeting the optimal nodes in one network structure may result in a larger improvements in spread than in other network structures. Identifying the network types in which GLIM is more efficient than other optimization algorithms and in which types not is relevant for potential implementation. Therefore, we evaluate the performance of GLIM in comparison to the outdegree and eigenvector centrality algorithms in various network types.

Network generators are algorithms that create artificial networks. We have selected six types of network generators that create networks with contrasting characteristics to test the performance. We evaluate directed/bidirected networks, networks with higher/lower connectivity, networks incorporating *preferential attachment* or a combination of these characteristics. *Preferential attachment* refers to the process in which new nodes are more likely to connect to nodes that already have more connections. Three types of directed graph generators described by Krapivsky and Redner [24, 25] are evaluated: Growing network (GN), growing network with redirecting (GNR) and growing network with copying (GNC). These generators all produce directed acyclic graphs (DAG), in which the GN network has the lowest connectivity and the GNC network the highest. In the GNR method, one must specify the probability of redirecting; in this paper *p* = 0.3 is arbitrarily chosen. A directed graph incorporating preferential attachment is introduced by Hansen and Jaworski [26–28]. In this paper a generalized version of this model, the random-*k* out graph [29] is used to evaluate the properties of the link-based influence maximization problem and to evaluate the performance of the proposed optimization algorithm. The random-*k* out graph produces directed graphs in with all nodes have *k* outgoing edges. Cycles can exist in this graph type.

The final network types we will evaluate are bidirectional networks. We consider two types: 1. tree networks with a power law (PL) degree distribution and 2. the Barabasi-Albert (BA) network, which is formed under preferential attachment [30]. Both network generator types produce undirected graphs. These are converted to directed graphs with bidirectional edges. In the Barabasi-Albert algorithm, networks are created by iteratively adding nodes that connect to a set amount of already existing nodes. One must specify the number of edges that every incoming node gets, *m*. In this paper this number is arbitrarily chosen as *m* = 5. A downside of evaluating a bidirectional network is that the number of simple paths increases dramatically, affecting also the computation time of the spread.

All tested networks consist of 50 nodes. We have chosen for the size of 50 nodes, because LIM is designed for health promotion programs and we assume that the networks in which LIM is applied are relatively small (<500 nodes). Example visualizations of all evaluated networks can be found in S1 Fig. The optimization takes place under an arbitrarily chosen fixed budget of *k* = 15. All weights of edges *w*(*e*_*v*_) are given value 1 and are normalized as 1/*d*^*in*^. The heuristics are compared on estimated influence during optimization and on computation time. All experiments are conducted on a 2016 MacBook Pro with 2.9 GHz Intel Core 5 with 16GB memory.

### Performance results of the algorithms

**Fig 3:**
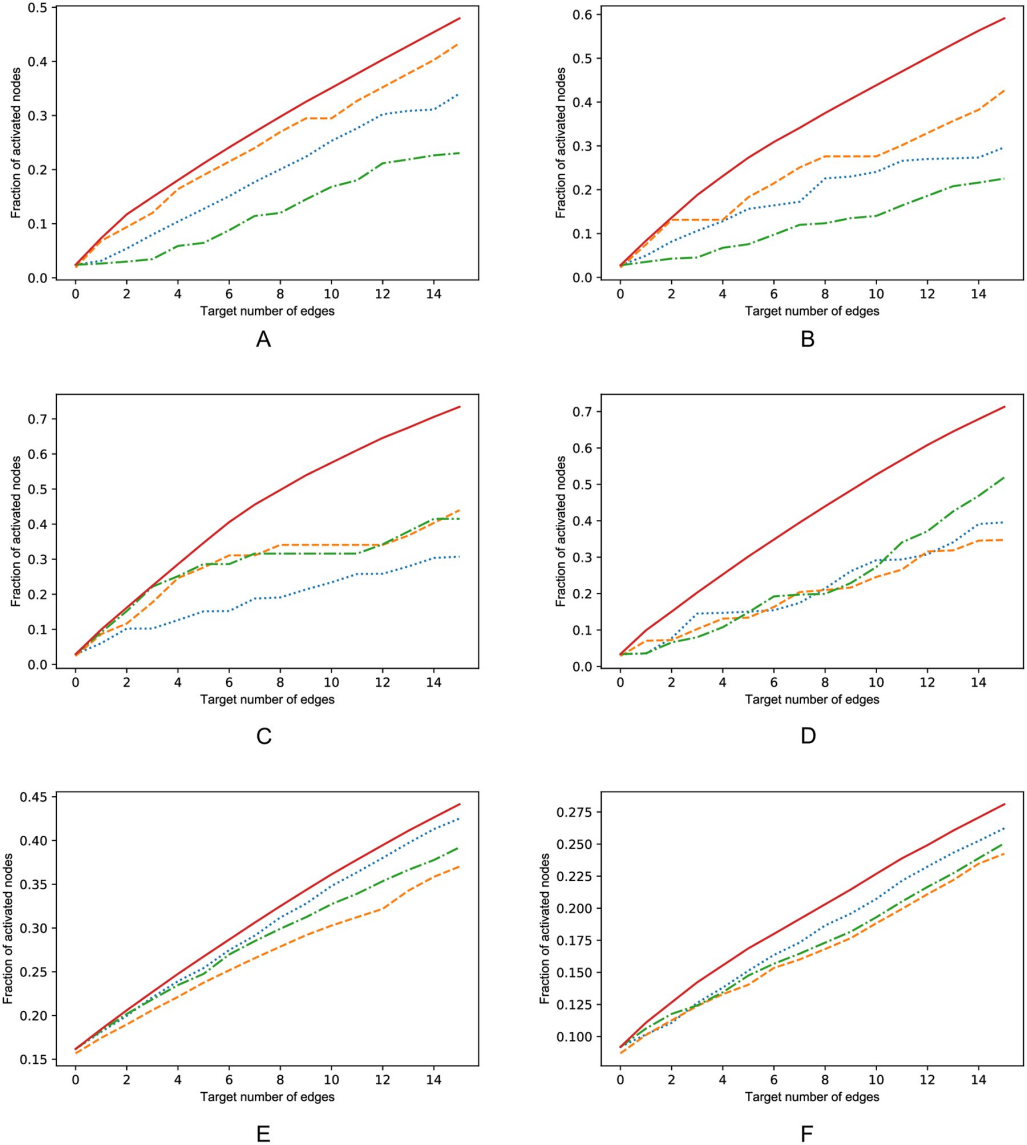
Performance of optimization algorithms in six different network types. On the x-axis the amount of selected edges is shown and on the y-axis the fraction of activated nodes. Network types are A: GN network. B: GNR network p = 0.3. C: GNC network. D: Random k = 5 network with 50 nodes. E: PL tree network. F: BA network m = 5. Legend: Solid red line: GLIM. Yellow dashed line: optimization using eigen vector centrality. Green dash-dotted line: optimization using outdegree. Blue dotted line: random edge selection.

The simulation results of the performance of the algorithm are shown in Fig 3. The random edge selection results serve as a baseline.

In all three directed acyclic graphs, GN, GNR and the GNC, GLIM outperforms the other heuristics. In the random *k* = 5 out network GLIM clearly outperforms the eigenvector and outdegree heuristics, while the eigenvector and outdegree heuristic perform only slightly better than random edge selection. Fig 3E shows the performance in the PL tree network. Notice that the network measures performed worse than the baseline random edge selection. Additionally, GLIM only slightly outperforms the baseline, implying that in this network structure the difference in increase between the optimal edge and a random edge is small. In Fig 3F the performance of the heuristics in the BA network is shown. GLIM outperforms the other heuristics, however the difference is not substantial.

The total spread resulting from optimizing LIM is different over the various network types. Targeting 15 nodes in the bidirectional networks of the PL tree and the BA network results in increases of the total influence to respectively 45% and 27.5% of the nodes, while targeting 15 nodes random-*k* = 5 out network leads to a total spread of approximately 70%. These results imply that the expected increase in spread and that the quality of the solution offered by GLIM are dependent on the network structure.

Table 2 shows the optimization time of the different heuristics in different network structures. The computation time of GLIM is considerably larger than the computation time of the alternative optimization strategies in all network types. In networks with higher connectivity estimating the spread with MC simulation is more demanding, increasing the optimization time. GLIM has significantly larger computation times in networks with high connectivity than in networks with low connectivity.

**Table 2:** Computation time (sec) in different network types under budget *k* = 15.

	Network type					
	Random *k* = 5 out	BA *m* = 5	PL tree	GN	GNR *p* = 0.3	GNC
Greedy	2955.40	2528.76	752.72	679.65	734.16	1904.78
Max degree	69.75	74.86	22.76	16.71	16.67	57.99
Eigenvector centrality	68.76	73.42	22.19	17.15	17.51	55.90

All simulations are done on networks of 50 nodes and the optimization constraint is *k* = 15 edges.

The results show that GLIM outperforms the alternative optimization algorithms in all network structures at the cost of higher computation times. Additionally, the results show that the performance of GLIM differs in different network structures. In bidirectional graphs (the PL tree and BA networks) GLIM only slightly outperforms random edge selection, while in directed graphs (the GN, GNR, GNC and random *k* out networks) it clearly offers advantages.

## Application: Dutch health promotion program

The link-based influence maximization problem (LIM) is developed for the specific case of maximizing the spread of health promotion programs. In this section we will apply LIM on a Dutch health promotion program and will compare the performance of GLIM algorithm in comparison to alternative optimization strategies on real data, to evaluate whether applying GLIM could help in formulating strategies. The performance of the algorithms is evaluated with the same methods as described in section Performance of optimization algorithms applied to LIM.

### Preventing obesity among children

Jongeren Op Gezond Gewicht (JOGG), translated as Children At Healthy Weight, is a Dutch national health promotion program that addresses overweight in children [31]. This program collaborates with municipalities to influence the environment of children aiming for a healthy lifestyle. To achieve behavioral change, JOGG reaches out to organizations in the proximity of children, such as schools and sport clubs, that can participate in the program. The schools and sport clubs form a network or organizations within the municipality. Then a local JOGG director is appointed, who is responsible for spreading the program, in this case the seed of the diffusion process. The JOGG director aims to maximize the participation of local organizations in the JOGG program in the municipality by approaching these organizations. This method corresponds with LIM: maximizing the spread by making new connections within an existing network. Optimizing LIM in a local network of objective organizations may help in creating strategies for JOGG.

### Data of JOGG

**Fig 4:**
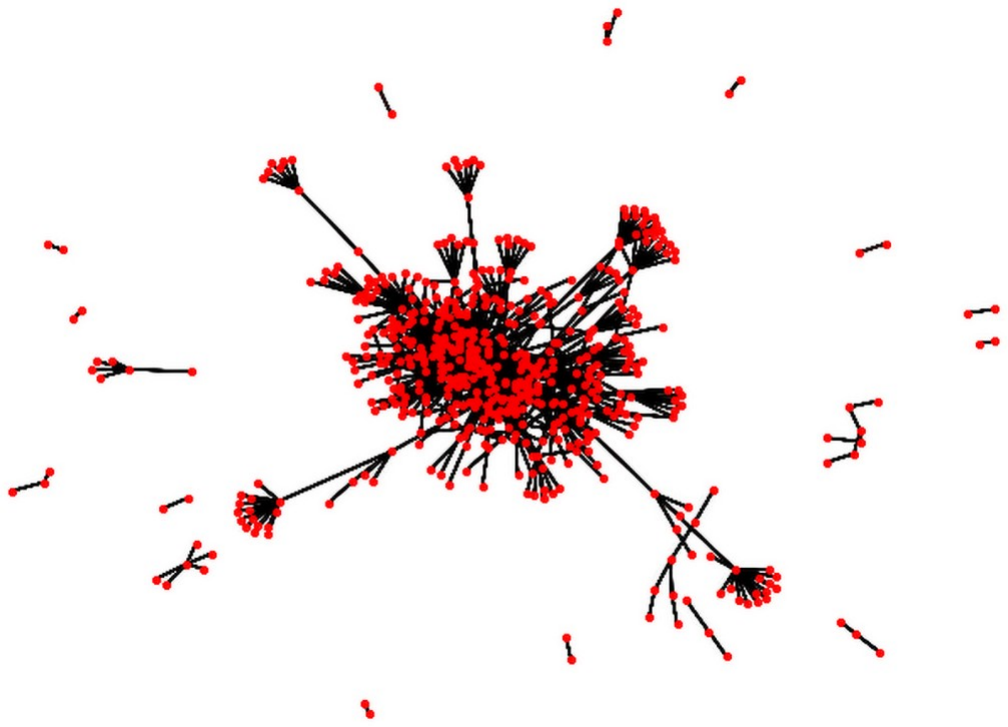
Example education network of an anonymous municipality. The red dots represent nodes and the black lines represent edges.

To evaluate the performance of GLIM in comparison to the alternative optimization algorithms in the setting of JOGG, we use network data from public organizations within a single municipality in the Netherlands. Target organizations operate in different sectors: education, sports, health and nutrition. Data was acquired from Statistics Netherlands. However, there is only limited data available on local networks in the Netherlands. Dienst Uitvoering Onderwijs (DUO), the Dutch government institution responsible for education, provided data that show the organizational relationships in education, such as relationships between holdings and subsidiaries and relationships between municipalities and educational organizations. Unfortunately, this data does not include collaborations, such as schools that organize events together. The DUO data only reflects the educational side of the public sector and does not include health or sports organizations which play a significant role in the spread of health promotion programs. In this paper the data from DUO of relations between education organizations in one anonymous municipality in the Netherlands is used as an example network and as a reference to evaluate the performance of the optimization algorithm, which will be referred to as the education network. Due to privacy legislation, we may not disclose the name of the municipality. Fig 4 provides a visualization of the network.

We evaluate the simulated performance of GLIM in comparison to the alternative optimization algorithms based on the heuristics outdegree and right eigenvector centrality as described in as described Section Performance of optimization algorithms applied to LIM. A random seed is chosen and the spread will be optimized under the arbitrary budget of 15.

### Results in JOGG

**Fig 5:**
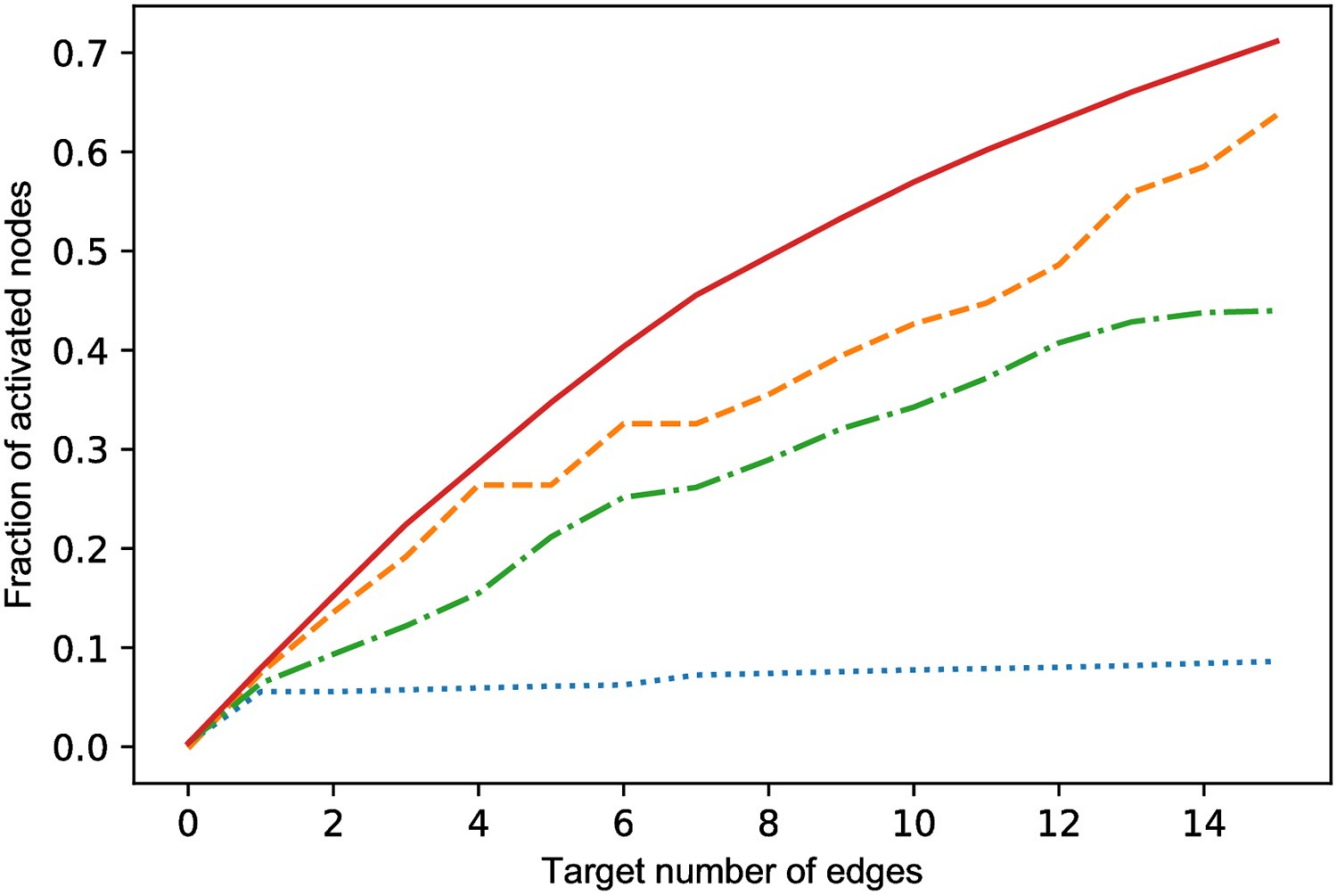
Performance of optimization algorithms in an network of education organizations of ± 250 nodes. On the x-axis the amount of selected edges is shown and on the y-axis the fraction of activated nodes. Legend: Solid red line: GLIM. Yellow dashed line: optimization using eigen vector centrality. Green dash-dotted line: optimization using outdegree. Blue dotted line: random edge selection.

The performance of the different optimization algorithms are shown in Fig 5. The random edge selection functions as a baseline. Clearly, the proposed greedy algorithm based on influence estimation outperforms the eigenvector and outdegree heuristics. The eigenvector centrality and outdegree heuristics perform significantly better than the random baseline in the education network.

The results regarding computation time are shown in Table 3. The computation time of random-*k* = 5 out network are shown in comparison. The computation time is considerably larger in the bigger education network of 250 nodes than in the network of 50 nodes. This implies that the proposed greedy algorithm is not scalable to larger networks.

**Table 3:** Computation time of the different heuristics in a generated network and in the Education network.

	Network size	
	Random *k* = 5 out 50 nodes	Education network ± 250 nodes
Greedy	2955.40 sec	42157.3 sec
Max degree	69.75 sec	175.6 sec
Eigenvector centrality	68.76 sec	180.3 sec

The optimization is done in both networks under a constraint of *k* = 15 edges.

GLIM outperforms the alternative optimization algorithms at the cost of higher computation times. Therefore, we believe that GLIM may help in formulating strategies for JOGG. The computation time of the algorithm is considerably larger in the larger network, so the advantage of GLIM may be more pronounced in smaller networks than in larger networks.

## Discussion

In this paper we propose a new influence optimization problem, the Link-based Influence Maximization Problem (LIM). The motivation for the name is that the action of the spreader trying to influence a node, can be viewed as creating a new (weighted) link between the two. We show that LIM has a monotonically increasing, submodular target function, allowing the use of a greedy algorithm for optimization. We explore the characteristics of LIM in the specific framework of health promotion programs. Several assumptions are made to provide theoretical proofs and to explore the global effectiveness.

First, in this paper we concentrate on the argument that the classic IMP is not suitable for maximizing spread in health promotion programs, due to the persuasion problem and the perspective problem as explained in the introduction. However, the unsuitability of IMP may extend to a more general premise. In reality, the persuasion assumption that an external party can choose a seed that adopts the product with absolute certainty is likely to be violated in many circumstances. Any product that cannot be provided free of charge, but instead requires some investment from the receiving party, might not be adopted with absolute certainty. The application of LIM can therefore be extended to any premise in which IMP is too “optimistic”. A concrete example in which LIM could be applied is: a new startup that aims to sell its product. The owners do not have funds for advertisements or for donating their product for free, but intend to use their personal network to spread the use of its product. LIM could help in finding which relations they should build to help spread the use of the product more realistically than IMP.

Second, in this paper we assume the linear threshold model is most applicable in the case of health promotion programs and show theoretical and simulation results only under this diffusion model. However, the linear threshold model may not be the most suitable diffusion model for all applications. For example the independent cascade model may be more applicable for word of mouth advertisement. It is not clear if the characteristics of monotonicity and submodularity hold under other diffusion models. Furthermore, the effectiveness of optimizing LIM may be different when using other diffusion models. Future research is needed to explore the characteristics of LIM in other diffusion models.

Third, we assume that the cost of every new link is equal. In reality this assumption may be violated, since it may be easier to connect with one person than to another. A logical extension of LIM may consist of including a cost function in the LIM greedy algorithm. Some cost functions that could be considered are: cost proportionate to distance, cost relative to the connected of nodes or cost corresponding to hierarchy. However, in this paper we use the submodularity of the LIM target function to provide an approximation guarantee. This approximation guarantee may not hold under cost functions. Future research is needed to explore the use of cost functions.

Fourth, all theoretical proofs in this paper require the assumption that the threshold distribution of the linear threshold random process follows a uniform distribution. It may be interesting to explore the characteristics of LIM under other threshold distributions.

This paper gives a first proposition of LIM. We propose to optimize LIM by using a greedy algorithm based on estimated influence, GLIM. Here we use Monte Carlo simulation to estimate the influence. This method is not scalable to larger networks. In the recent literature many fast influence estimation and influence optimization algorithms have been described for IMP. Some of these methods may be used to develop more efficient optimization algorithms for LIM,for instance by using Reverse Influence Sampling [20–22].

Finally, in this paper we show by example that LIM provides different results than IMP. We analyzed the performance of GLIM within different network types, but we did not evaluate the topological characteristics of the nodes provided by GLIM in detail. It may be interesting to further evaluate the (differences in) results provided by LIM and IMP from both a performance and topological perspective.

### Conclusion

Due to rising health care costs, the need for cost-effective health promotion programs is high and alternative approaches to improve of these programs are crucial. In this paper, we propose an innovative computational approach to improve the effectiveness of health promotion programs. We argue that the classic Influence Maximization Problem (IMP) is unsuitable for modeling the maximization of spread in health promotion programs, due to the persuasion problem and the perspective problem. We propose a “Link-based Influence Maximization Problem” (LIM) by changing the decision variable of the spreader from choosing an optimal seed to selecting an optimal node to influence.

A greedy algorithm based on estimated influence, GLIM, for optimizing LIM was developed. We compare GLIM to alternative greedy algorithms using common network measures outdegree and right eigenvector centrality in different network types and in real data of a Dutch health promotion program, to identify in which network structures GLIM has potential use. We show that the quality of optimization strategies depends heavily on the network structure. The gain in total spread is substantially higher in unidirectional networks than in bidirectional networks. Finally, we show that GLIM outperforms the alternative algorithms in all simulations and network types and always results in the highest estimated total spread at the cost of a higher computation time. Therefore, we believe that applying GLIM may help in formulating strategies for maximizing the spread of health promotion programs and other circumstances in which IMP is unsuitable with the aim to improve the (cost)effectiveness.

## Supporting information

S1 Appendix Example IMP vs LIM. (PDF)

S1 Fig Visualizations of networks created by different network generators. A: Growing network. B: Growing network with redirecting. C: Growing network with copying. D: Random *k* = 3 out network. E: Power-law tree. F: Barabassi-Albert network.
(TIF)

S1 File Code and data used in this paper. (ZIP)
