# Supplementary material for: Link-based influence maximization in networks of health promotion professionals
Source: PLoS One. 2021 Aug 25;16(8):e0256604. doi: 10.1371/journal.pone.0256604 (PMC8386878; doi:10.1371/journal.pone.0256604)
Supplement: S1 Appendix — (PDF) [file pone.0256604.s001.pdf]

S1 Appendix Example IMP vs LIM

This is an example of the greedy algorithm in IMP versus LIM. Here, we show the exact calculations used to arrive at the conclusions shown in section Formal definition of LIM. Consider the graph of 5 nodes, $\{s, 1, 2, 3, 4\}$ as depicted in Fig 6. We will perform the greedy algorithm as described in section Performance of optimization algorithms applied to LIM, but instead of using Monte Carlo simulation we will use exact spread calculation using the closed formula as given by Chen, Yuan and Zhang [1], $\sigma(\cdot)$ for $\mu = \text{unif}[0, 1]$:

$$\sigma(\cdot) = \sum_{\pi \in \mathcal{P}} \prod_{e \in \pi} w_n(e)$$

By definition the path only containing s has weight 1. In the LIM the $\sigma(\cdot)$ is influenced in two ways: 1. The amount of simple paths change, 2. the weights change. In this example we assume the case where all incoming edges are weighted equally and then normalized.

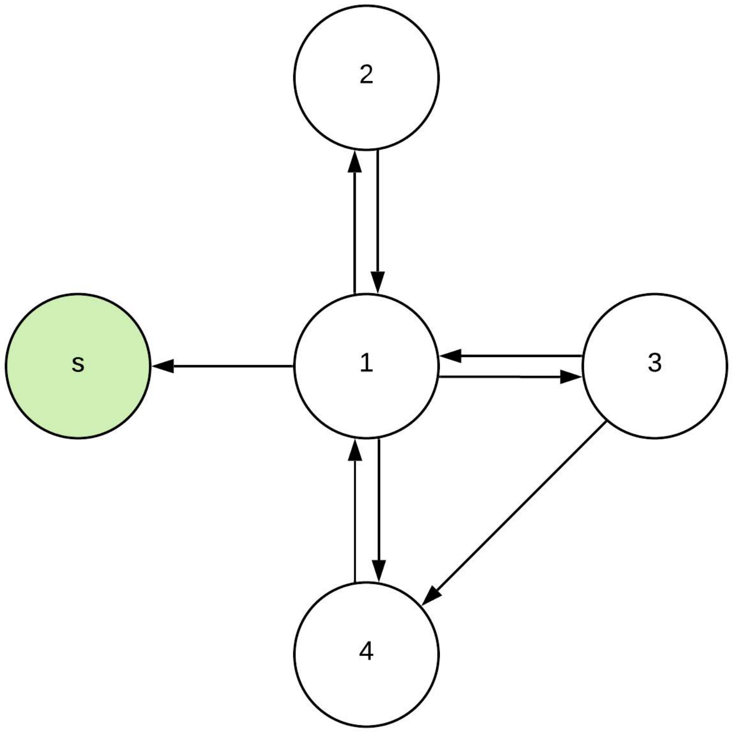

Fig 6. Example network The circles represent nodes, in which the green circle is the spreader. The arrows represent edges.

First we will show the selection procedure in the classic IMP.

$$\begin{aligned}
\sigma(\emptyset) &= \sum_{\pi \in \mathcal{P}} \prod_{e \in \pi} w(e) && = 1 \\
\sigma(\{1\}) &= 1 + 1 + 1 + 1 + 1 * 0.5 + 0.5 && = 5 \\
\sigma(\{2\}) &= 1 + 1 + 1/3 + 1/3 * 0.5 + 1/3 * 0.5 && = 3 \\
\sigma(\{3\}) &= 1 + 1 + 1/3 + 1/3 * 1 + 1/3 * 0.5 + 0.5 + 0.5 * 1/3 + 0.5 * 1/3 * 1 && = 3.5 \\
\sigma(\{4\}) &= 1 + 1 + 1/3 + 1/3 * 1 + 1/3 * 1 && = 3
\end{aligned}$$

The greedy algorithm will select node 1.

Now we will show the selection procedure in LIM:

$$\begin{aligned}
\sigma(\emptyset) &= \sum_{\pi \in \mathcal{P}} \prod_{e \in \pi} w(e) && = 1 \\
\sigma(\{1\}) &= 1 + 1/4 + 1/4 * 1 + 1/4 * 1 + 1/4 * 1 * 0.5 + 1/4 * 0.5 && = 2 \\
\sigma(\{2\}) &= 1 + 0.5 + 0.5 * 1/3 + 0.5 * 1/3 * 1 + 0.5 * 1/3 * 1 * 0.5 + 0.5 * 1/3 * 0.5 && = 2 \\
\sigma(\{3\}) &= 1 + 0.5 + 0.5 * 1/3 + 0.5 * 1/3 * 1 + 0.5 * 1/3 * 0.5 + 0.5 * 0.5 \\
&\quad + 0.5 * 0.5 * 1/3 + 0.5 * 0.5 * 1/3 * 1 && = 2\frac{1}{3} \\
\sigma(\{4\}) &= 1 + 1/3 + 1/3 * 1/3 + 1/3 * 1/3 * 1 + 1/3 * 1/3 * 1 && = 1\frac{2}{3}
\end{aligned}$$

The highest spread is gained from choosing node 3.

References

1. Chen W, Yuan Y, Zhang L. Scalable Influence Maximization in Social Networks under the Linear Threshold Model. In: 2010 IEEE International Conference on Data Mining; 2010. p. 88–97.
