# Supplementary figures and images for: Link-based influence maximization in networks of health promotion professionals
Source: PLoS One. 2021 Aug 25;16(8):e0256604. doi: 10.1371/journal.pone.0256604 (PMC8386878; doi:10.1371/journal.pone.0256604)

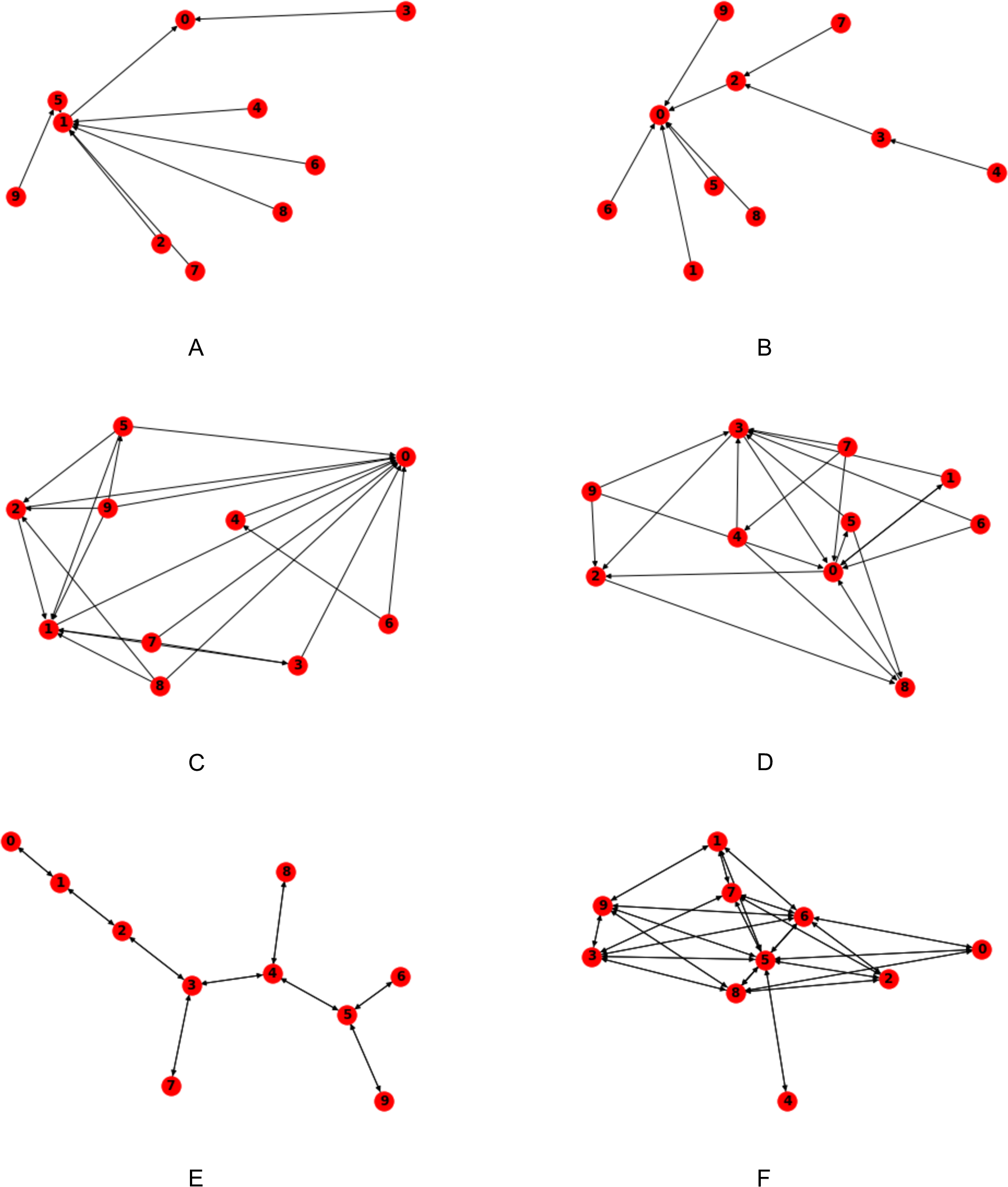

Supplement: S1 Fig — A: Growing network. B: Growing network with redirecting. C: Growing network with copying. D: Random k = 3 out network. E: Power-law tree. F: Barabassi-Albert network. (TIF) [file pone.0256604.s002.tif]
